# The state of macro-energy systems research: Common critiques, current progress, and research priorities

**DOI:** 10.1016/j.isci.2023.106325

**Published:** 2023-03-05

**Authors:** Rachel Moglen, Kiran Prakash Chawla, Patricia Levi, Yinong Sun, Oladoyin Phillips, Benjamin D. Leibowicz, Jesse D. Jenkins, Emily A. Grubert

**Affiliations:** 1 Operations Research and Industrial Engineering, The University of Texas at Austin, Austin, TX 78712, USA; 2 Stanford Law School and Emmett Interdisciplinary Program in Environment and Resources, Stanford University, Stanford, CA 94305, USA; 3 Form Energy, Berkeley, CA 94710, USA; 4 Environmental Health and Engineering Department, Johns Hopkins University, Baltimore, MD 21205, USA; 5 School of Public and International Affairs Center for Policy Research on Energy & Environment and Andlinger Center for Energy and the Environment, Princeton University, Princeton, NJ 08544, USA; 6 Department of Mechanical and Aerospace Engineering and Andlinger Center for Energy and the Environment, Princeton University, Princeton, NJ 08544, USA; 7 Keough School of Global Affairs, University of Notre Dame, Notre Dame, IN 46556, USA

**Keywords:** Energy policy, Energy systems, Energy management, Energy modeling

## Abstract

The growing field of macro-energy systems (MES) brings together the interdisciplinary community of researchers studying the equitable and low-carbon future of humanity’s energy systems. As MES matures as a community of scholars, a coherent consensus about the key challenges and future directions of the field can be lacking. This paper is a response to this need. In this paper, we first discuss the primary critiques of model-based MES research that have emerged because MES was proposed as a way to unify related interdisciplinary research. We discuss these critiques and current efforts to address them by the coalescing MES community. We then outline future directions for growth motivated by these critiques. These research priorities include both best practices for the community and methodological improvements.

## Graphical abstract

**Figure:**
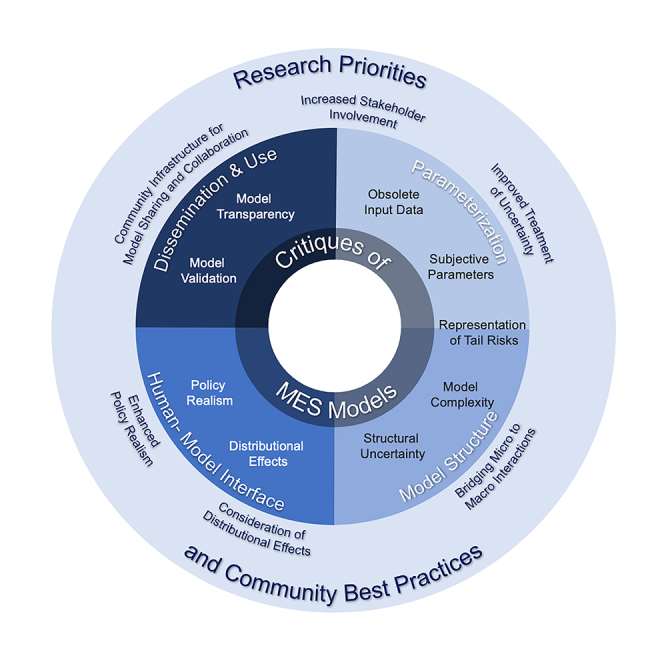

## Introduction

Today’s energy systems, from fuel to electricity, face the interrelated and pivotal challenges of climate change, a growing global energy demand, and a need for more equitable access. Decarbonization of the energy sector through the transition to low- and zero-carbon energy sources is central to mitigate the threat of climate change.^1^ Energy systems must also expand to meet energy demands from a growing global population. Simultaneously, energy systems need to improve the services provided to those living with limited energy access. How humanity chooses to address these challenges will drive fundamental changes in human, economic, and environmental systems in the coming decades. The growing research field of macro-energy systems (MES) is poised at the forefront of this movement, developing and applying new methods for the study of complex energy systems to improve energy policy and decision making.

MES brings together researchers across many disciplines including engineering, economics, public policy, and other social sciences, each bringing methodological tools and research perspectives unique to their respective disciplines. Because MES is a methodologically diverse field, in this paper we focus on the branch of MES research that relies on quantitative modeling. MES unites the diverse community of researchers who are studying human energy systems at large scales.^2^^,^^3^ The problems and systems addressed by MES involve massive scales temporally (years), spatially (km^2^), and energetically (GWh). Any one of these dimensions is sufficient to define a problem and an energy system large enough to be considered MES research.^2^ An inaugural workshop^4^ on the topic of MES identified the following research questions as central areas of study in MES.

1. How can we expand energy access affordably and sustainably?
2. How will policy affect the evolution and use of energy systems?
3. What technology portfolios do we need for climate-friendly energy systems?
4. How will energy systems affect environmental and human systems?

Although MES has only recently begun to coalesce as a well-defined research community, some of the research it brings together has existed for many years across different disciplines and research communities. Historically, communities in the energy-climate-sustainability space have been defined by specific modeling tools or research practices (e.g., integrated assessment modelers, energy systems modelers using tools like TIMES and OSeMOSYS, and lifecycle assessment researchers). MES is a community with more fluid boundaries, unified by topic rather than a specific methodology or discipline. This creates an environment that makes it easier to share ideas, methodologies, and perspectives.^3^ Often, a pathway for addressing critiques leveled at a certain branch of MES can be found in the work conducted by other disciplines within MES. For example, the current MES optimization literature increasingly acknowledges the importance of objectives beyond cost-minimization when developing portfolio recommendations for “optimal” energy systems,^5^ including distributional effects that have long been a focus of the social science MES literature.^6^ Other examples include collaborations between modelers and public policy researchers to improve policy realism in energy models.^7^^,^^8^

The goal of this paper is to guide the growing MES community through a better understanding of the key obstacles and questions facing the field, as well as the state of the art in existing methodologies and results. Because MES research exists across many professional communities, conferences, and journals,^3^ it is often siloed by discipline or methodology. Without the typical support of established, focused journals or unifying conferences to facilitate communication across the community, it can be challenging for MES researchers to identify common critiques and the corresponding research priorities. Pitfalls of this lack of communication include research that “reinvents the wheel” or does not take advantage of and build on existing methodologies. These inefficiencies are especially undesirable given the scale and urgency of the climate crisis, and the role of MES research in supporting a sustainable transition. This paper draws on feedback from researchers from across the MES arena who participated in two workshops on this emerging field, the first in the fall of 2020 and the second in spring 2022.^4^ Specifically, this paper was initially inspired by two of the first workshop’s panels: “Critiques of Macro-Energy Systems Research and Our Responses as a Field” and “Frontiers of Macro-Energy Systems Research.”

The remainder of the paper is organized as follows. First, we summarize some of the critiques of MES research as well as ongoing efforts to address these critiques, synthesizing this paper’s observations in a succinct tabular format. Then, we lay out a blueprint for promising research directions for the community, and how these research priorities address common critiques of MES research. We conclude by summarizing critiques of the field and high-priority future research directions.

## Critiques and current progress

Research themes in MES have evolved over the last few decades from a focus on technical feasibility and cost minimization to multi-attribute analyses of decarbonization pathways and implementation challenges. Despite significant progress in the field, a number of critiques of the models, methods, processes, and assumptions frequently employed by MES researchers have been raised that need to be addressed more thoroughly. These critiques may not be unique to MES research, and may apply only to a subset of the diverse work being done by the community. The following subsections each discuss a specific critique of MES research and current research efforts that engage the criticisms. Table 1 summarizes each of the detailed critiques in the following sections and identifies key references that either discuss or directly address the critique.

**Table 1:** Critiques of MES research

Critique	Summary	Discuss Critique	Address Critique
**Model Validation**: MES models are not subjected to rigorous validation, or that such validation is impossible.	• Validation traditionally concerns testing a model for precision and accuracy • Validation of the large-scale forward-looking models is difficult or impossible, has been a challenge for similar fields as well • Validation should be considered holistically with the other components of modeling exercises	Parker et al.^9^, Kling et al.^10^	Bennett et al.^11^, Jakeman and Letcher^12^, Laniak et al.^13^, Chaturvedi et al.^14^
**Subjective Parameters**: Some crucial model inputs are subjective or arbitrary.	• These inputs are necessary because they represent value-judgements or reflect deep uncertainty, but may vary across actors • Can be addressed through transparency about assumptions, and sensitivity analysis • For example, selecting the discount rate is a subjective decision with significant impacts	Kling et al.^10^, Pindyck^15^, Vale^16^, Dasgupta^17^, Risbey et al.^18^	Dasgupta^17^, Kann and Weyant^19^, Giglio et al.^20^
**Model Complexity**: MES models are usually either too complicated or too simple for their application.	• The level of complexity and detail in an MES model or framework is a key choice • Approaches may be too simple and high-level to produce the information that is needed for local decision support, or they may be too complicated and detailed to yield generalizable insights • It is important for approaches to be adaptable to the level of detail called for in the situation	Fisher-Vanden and Weyant^21^	Priesmann et al.^22^, Jenkins and Sepulveda^23^, West et al.^24^
**Obsolete Input Data**: Parameters are changing so fast that even the most up-to-date versions of models include obsolete input data.	• Fast-changing data include technological advances like solar PV and battery costs, policy changes like a carbon tax, and behavioral changes • Rapid evolution of these parameters threatens to undermine research conclusions • Solutions include sensitivity analysis, transparency, and more efficient data pipelines	Berckmans et al.^25^, Bullard and Johnson^26^, Robertson^27^	Krey et al.^28^, Markov et al.^,^^29^
**Policy Realism**: MES work does not feature enough policy, institutional, and behavioral realism.	• Analysis tends to focus on high-level policy instruments rather than the more granular policies that tend to be implemented • Real world policy implementation occurs through a suite of policy instruments • MES needs to account for institutional and political realities of implementation	Grubert^30^, Gambhir et al.^31^, Weyant^32^, Peng et al.^33^	Zhu et al.^7^, Peng et al.^33^, Park and Baldick^34^, Palmer and Burtraw^35^, Battiston et al.^36^, McCollum et al.^37^, Cullenward and Victor^38^
**Distributional Effects**: Models have not focused enough on the distributional effects of climate change and climate policies.	• MES researchers historically have focused on technical and financial feasibility and have neglected equity concerns and implementation challenges • Recent shifts in MES research toward multi-objective and multiple-alternatives approaches, as well as distributional and political economy issues • MES modelers should increase collaboration with other MES researchers who have studied these issues historically, such as social scientists	Weyant^32^, Caron et al.^39^, Fortes et al.^40^	Pachauri et al.^41^, Mastrucci et al.^42^, Raimi et al.^43^
**Model Transparency**: There is a lack of transparency regarding model structures, perspectives, and assumptions. As a result, it would be virtually impossible for people other than the model developers themselves to reproduce results.	• MES analyses are often criticized for lack of transparency • Part of this critique is a process issue (i.e., people and perspectives are excluded from model development on an accessibility basis) • Ongoing efforts to address this critique include broader involvement in model development, documentation, open source models, model tutorials, and publication of outputs for general use	Risbey et al.^18^, Gambhir et al.^31^, Bistline et al.^44^, Bazilian et al.^45^, DeCarolis et al.^46^	Bistline et al.^44^, Bazilian et al.^45^, DeCarolis et al.^46^, Middleton et al.^47^, Pfenninger et al.^48^, Grubert^49^
**Structural Uncertainty**: Representation of uncertainty in MES models has focused too narrowly on parametric as opposed to structural uncertainty (e.g., growth rates, substitutable damages, utility functions).	• Structural uncertainty concerns choices like the boundary of the analysis, abstractions used to represent complex realities, and how different system components interact with each other • Scenario analysis, modeling to generate alternatives, and model intercomparison have been used successfully to illustrate the effects of structural choices • There is significant room to enhance our use of uncertainty methods here and more broadly	Krey et al.^28^, Weyant^32^	Priesmann et al.^22^, Knopf et al.^50^, DeCarolis et al.^51^, Gillingham et al.^52^
**Tail Risks**: Models often neglect tail risks such as catastrophic climate change, even though this might be a primary determinant of optimal decision making.	• Structural assumptions about distributions in combination with risk aversion can have a significant impact on decision relevant recommendations • Negative impacts of climate change, extreme weather events and, on the upside, technological breakthroughs are key examples	Pindyck^15^, Ghambhir et al.^31^, Kousky and Cooke^53^, Weitzman^54^, Nordhaus^55^	Nordhaus^55^, Baik et al.^56^, Sepulveda et al.^57^, Cai and Lontzek^58^, Greenstone et al.^59^, Heuberger et al.^60^

### Model validation

Model validation is the assessment of model accuracy through comparison to independent observations, and can be a critical component of the modeling process. Fundamentally, model validation presents a challenge to forward-looking MES models,^9^^,^^10^ given the absence of independent future observations for comparison. Available data may not be representative, or fundamental changes may be expected, changing the dynamics of the system.^11^ Climate prediction, an area adjacent to MES, faces similar validation difficulties driven by the absence of data describing the future. Climate models are typically validated via ‘backcasting’ or ‘hindcasting’ by initializing based on historic conditions and projecting forward to determine how well modeled outcomes compare to recent historical observations.^61^^,^^62^ However, the challenge can be greater for MES models which include human behavior and economic and policy decision making. These decisions are far less likely to obey immutable laws as do purely physical systems modeled in the physical sciences. Furthermore, an MES backcasting exercise would need to know the precise set of information each decision maker was privy to at the time their decisions were made, an epistemically impossible task. Overall, MES research is often critiqued for lack of validation, reflecting the concern that conclusions found by the MES community are inaccurate, or that the level of accuracy is unknown.^10^

Although model validation can be an important modeling step, it is only a subset of the broader model evaluation process. Model evaluation quantitatively and qualitatively determines a model’s applicability and efficacy through exploration of its uncertainties.^13^ Model evaluation can include model accuracy assessment through backcasting, uncertainty quantification, transparency, risk analysis, communication with stakeholders, validation of physical or engineering constraints, and exploration of system insights.^12^^,^^13^^,^^14^ All these activities are customary practice in MES modeling.^11^^,^^12^^,^^13^^,^^14^ Although MES models may be justly critiqued for lack of validation, given the inaccessibility of future data against which to validate, other evaluation methods are employed beyond validation. Finally, it is important to remember the context in which the models are used: the goal of MES models is to provide decision support which may not require perfect accuracy.

### Subjective parameters

Parameterization of MES models involves selecting inputs that may be subjective, value-laden, or arbitrary.^10^^,^^15^^,^^16^^,^^18^ These inputs are necessary because they represent relevant value-judgments that must be considered when making choices related to our energy systems. Nevertheless, putting a number to ideas like the dollar value of a human life saved, the value of future generations relative to the present generation (i.e., the discount rate), or the risk aversion of a person or a society, is inherently fraught and subjective.^17^^,^^19^^,^^20^ Other input parameters are fundamentally unknowable, such as the future cost and availability of nascent technologies or government policies decades in the future. Furthermore, different parties may not agree on the values for such parameters, both within the research community and among decision makers. These parameters and assumptions become a problem particularly when there is not an explicit acknowledgment of this subjective choice or adequate sensitivity testing of the impact that subjective parameters may have on the results.

Although MES models will always require subjective parameters as inputs, critiques about their selection can be mitigated through transparency about modeling assumptions. The importance of model transparency is discussed later in more detail, where subjective input parameters are one of the motivating cases. In addition, sensitivity analysis can be used to illustrate the role and importance of the assumptions underlying the selected parameters. This approach can indicate how subjective modeling decisions may be driving results and identify the need for further scenario analyses.^46^ Finally, incorporating the perspectives of a greater diversity of researchers and stakeholders can help parameterize subjective inputs and help clarify their implications for policy- and decision-making.^7^ Thus, although the critique of subjective input parameters cannot be entirely addressed, it can be mitigated through model transparency and sensitivity analysis.

### Model complexity

The level of complexity in MES models is a choice that has important impacts for how the resulting analysis can be used.^21^ A more abstract model may be solved analytically to provide generalizable insights or may be more computationally tractable, enabling much broader uncertainty analysis. Conversely, more detailed models capture more realism with the detail they include. These details include nuances of system operation, types of policy interventions, spatial and temporal resolution, and interactions both within the modeled system and between it and the broader environment. Choosing the right amount of detail for the application in question is a recurring quandary for modelers in MES; in a field where abstraction is a defining aspect of the methodology, how much abstraction should be used^22^?

Several approaches seek to mitigate this critique by matching the level of detail in the model to its use case. Some tools under development, such as the GenX electricity system planning model,^23^ have built-in abilities to fine-tune and change the level of detail instead of requiring the researcher to switch to an entirely different modeling tool to achieve a different level of resolution. Another approach involves starting with high-level, abstract output and downscaling to investigate results at the local level.^24^ Downscaling approaches produce granular output suited to the needs of local decision makers. Finally, deeper involvement of stakeholders in the modeling process ensures that MES models have the level of complexity that they need to answer the questions posed by decision makers. Stakeholders include policy makers and the people impacted by the decisions they make. Overall, the role of stakeholders in MES research is an area of future work and growth for the field, as referenced in the section titled “Increased stakeholder involvement”. Going forward, balancing the trade-off between model complexity and abstraction will be an open area of study in the MES community.

### Obsolete input data

Modeling rapid change is an ever-present challenge when working in a field with active progress.^31^ Technological, policy, and behavioral change can be captured either as endogenous processes that evolve with time in response to other model features or as exogenous input parameters; the latter case is discussed in this section. In MES, changing input data include technology costs like solar PV and battery costs driven down by technological advancement, policy changes like a carbon tax, and behavioral changes like environmental awareness.^25^^,^^63^^,^^64^ The speed with which these parameters change can mean data and the associated modeling results that are only a few years old may be already obsolete.

Modeling rapidly changing parameters remains an open challenge to the MES community. Because these input parameters are frequently exogenous to MES models, modelers must routinely update their data inputs to ensure validity of their results. Technological advances in data management, like sensors and improved data accessibility, can help streamline the data pipeline from source to model.^29^ Modelers also often employ sensitivity analysis to address rapidly changing input data. By varying input data as a sensitivity analysis, modelers can investigate the robustness of results to parametric perturbations, investigating the validity of results across the uncertainty of future change.^28^ Finally, model transparency can help mitigate this critique: by clarifying where data come from, the user can note outdated sources and interpret model results with that perspective.^27^

### Policy realism

The policy realism critique can be discussed in two parts: 1) the realism of policy instruments represented in MES models, and 2) the institutional, behavioral, and political realities of policy implementation. First, MES models often analyze broad policy instruments such as economy-wide emissions limits or uniform carbon prices when in reality, policy implementation occurs through a suite of policy instruments such as portfolio standards, low-carbon fuel standards, technology mandates, and specific subsidies.^38^^,^^65^ MES models often do not represent these more granular policy instruments, meaning that analyses of them are rare despite their appeal in real-world policy settings. Second, some MES models may not account for political and institutional realism such as the real-world feasibility or political appetite for different decarbonization pathways. In addition, the values held by different populations (i.e., social context) influence how environmental impacts are actually experienced.^30^ Incorporating these realities into MES frameworks has not been a norm. Finally, MES models are generally constrained to policy instruments with some history: as with all empirically grounded modeling there is a bias toward existing structures.

The MES community has been addressing these critiques in multiple ways. First, some studies directly compare and contrast the relative merits and costs associated with different policy instruments to provide a clear picture of trade-offs associated with pursuing one instrument over the other.^7^^,^^34^^,^^35^^,^^66^^,^^67^ Second, studies have attempted to highlight the importance of institutional and behavioral dimensions for decarbonization. Works that demonstrate how accounting for these dynamics can meaningfully affect decision-relevant conclusions for energy system transitions include investigations of market structure and industry competition,^68^ expectations and beliefs of investors,^36^ and technology adoption by individuals.^37^ Explicit collaboration with political scientists, behavioral psychologists, and others who study these areas can also improve the realism of MES models.^33^ Lastly, MES modelers can address the policy realism critique by clarifying the role of their recommendations in policy analysis. Emphasizing any institutional, behavioral, or political assumptions allows policy makers to choose what is realistic for them.

### Distributional effects

Climate change and climate policies do not impact the population uniformly. Those least responsible for climate change will experience the worst of its impacts while having the most limited ability to adapt. In their work, MES modelers routinely make assumptions about the key metrics for defining the “best” climate policies. Because until recently MES modelers focused on the technical feasibility of decarbonization pathways, the absence of justice considerations from MES models could lead to policy recommendations that disproportionately disadvantage certain demographics while still minimizing financial costs. Furthermore, MES modelers must recognize that we are not starting from a place of equity. Therefore, any forward-looking efforts to include distributional considerations in MES models will still result in significant injustice if existing inequities are not corrected.

The MES community is actively working to address this critique through methodological and structural changes to their models. First, some MES energy modelers are shifting from cost minimization objectives to multi-criteria decision analysis (MCDA) and modeling to generate alternatives (MGA) techniques.^69^ MCDA techniques allow researchers to explicitly explore trade-offs between energy decarbonization pathways along the dimensions of interest such as costs, equity, land use, and water use. Similarly, MGA methods produce a range of alternative pathways with similar costs but different equity and implementation implications, allowing decision makers to pursue alternatives most suited to their particular decision context. In addition, MES researchers have begun including distributional impacts in their models through a number of methods. Some authors explicitly highlight the distributional impacts of different policy packages.^39^^,^^70^ Others demonstrate the implications of meeting equity goals and potential pathways.^41^^,^^42^ Finally, another branch of MES research has begun to incorporate qualitative socio-economic storylines alongside quantitative narratives to provide a full picture of future energy scenarios.^43^

### Model transparency

A lack of transparency can make it difficult for researchers inside and outside the MES community to interpret, reproduce, and build on MES models. Model transparency can be improved by making source code available, publishing input and output data, documenting equations used, offering user training, and clearly discussing value-laden assumptions.^44^^,^^45^^,^^46^^,^^49^ Although publishing code and data are becoming increasingly common best practices in the MES community, Bistline et al.^44^ found several case studies where lack of transparency in value-laden assumptions led to significant impacts. In these cases, code and data availability alone were insufficient to ensure model transparency for stakeholders. Bazilian et al.^45^ similarly discuss the need for transparency best practices beyond code and data availability; they identify barriers that make it difficult or even prevent users from adopting and adapting the open MES models. Furthermore, this critique demonstrates the need for deeper involvement of diverse perspectives in model development.^49^ Stakeholders, particularly those impacted by decisions influenced by the model, should be included in the modeling processes from inception and experimentation through the presentation of results. Overall, MES will progress more rapidly with improvements in model transparency coupled with stakeholder engagement as the foundation for further progress.

Although the MES community has made improvements in model transparency, primarily in the form of code and data availability directed at other modelers,^44^ there is still room for growth. Ongoing efforts to address the critique of model transparency include model documentation, the model-sharing initiatives, model tutorials and community trainings, increased stakeholder involvement, and publication of model output for general use. Models such as SimCCS^47^ have detailed documentation, though at times this documentation assumes an audience inside the MES community, making it challenging for non-traditional MES researchers to access and implement MES models. Model-sharing initiatives include openmod^48^ which seeks to “advance knowledge and lead to better energy policies” through “open models and open data.” Tutorials and workshops hosted at conferences or independently also serve to teach prospective MES researchers to use MES models, often with simplified and accessible model versions. However, work remains to be done to both make these tutorials available to a broader audience and involve stakeholders throughout the modeling process.^45^ Finally, acknowledging that not all those interested in MES are modelers, the MES community sometimes releases model outputs for those wishing to interact with the data but not to implement the models themselves, but as cautioned by DeCarolis et al.,^46^ this practice should include communication of assumptions and limitations so that users do not incorrectly interpret the data.

### Structural uncertainty

MES modelers use abstract representation to model real-world processes and their interactions. The complexity and scale of systems studied in MES require ubiquitous abstraction, which generates many structural choices about how the model represents reality. Common structural decisions in the abstraction process include preferences (i.e., utility functions), growth rates, and substitution effects. Structural uncertainty is uncertainty in the functional forms used to represent the real underlying phenomena. Historically, the MES modeling community has focused less on structural uncertainty than on parametric uncertainty, which is uncertainty in the specific values used to parameterize MES models. For example, the cost of a solar panel in a model is a parametric uncertainty, whereas the form of an individual’s utility function is a structural uncertainty. Typical approaches to addressing parametric uncertainty, like Monte Carlo simulation analysis, decision making under uncertainty, and robust decision making, often are not used to address structural uncertainty. Structural uncertainty complicates comparing model results, as discussed by Krey et al.^28^

Although MES has begun to address structural uncertainty, there remains room for progress within the community. Scenario analysis and model comparison have been used successfully to illustrate the effects of structural choices.^22^^,^^50^ MGA is another approach gaining traction that does not explicitly represent different model structures but can give some insight into how sensitive outputs may be to structural changes.^51^ Other work employs structured model inter-comparisons aimed at isolating structural uncertainty across models.^52^ Further complicating the challenge of structural uncertainty, lack of model transparency makes comparison of model assumptions and structure more challenging, as discussed in the section titled “Model transparency”. Krey et al.^28^ therefore suggest that by improving model transparency through documentation, modelers may also help address the issue of structural uncertainty.

### Tail risks

Though rare, the risks resulting from unlikely events can be a primary factor in determining the performance of a candidate solution. However, MES research sometimes fails to appropriately account for these tail risks.^55^ In particular, this critique has been raised in the context of catastrophic climate events,^15^^,^^53^^,^^54^ especially because structural assumptions about risk tolerance and the distributions of uncertain parameters can have a significant impact on decision-relevant findings. Conversely, neglecting tail opportunities can contribute to an underestimation of cost-effective energy transition strategies emerging from technological breakthroughs or other positive tail effects. Although tail opportunities apply to a broad range of modeling efforts, they are particularly crucial for energy-focused models. For example, Heuberger et al.^60^ explore the impact of a potential ‘unicorn technology’ on total power system costs over a 35-year time horizon, finding that a revolutionary innovative technology could reduce total costs by 13%. Overall, tail opportunities may be one of the biggest drivers of costs and cost-optimal portfolios.

Historically, modelers have excluded tail events from their analysis because of the challenge of specifying the distribution of climate damages and the sensitivity of results to the assumed distributions^55^ and the difficulty of predicting revolutionary technological developments. Despite these challenges, several studies try to capture how positive tail effects and innovation may impact decision making. For example, Bollinger and Gillingham^71^ and Leibowicz^68^ both quantify how innovation may affect technology prices because of market structure and learning-by-doing effects. Other studies explicitly account for the impact of directed innovation on green technologies and the long run effect on emissions.^72^ MES modelers are also increasingly assessing the negative impacts of tail risks in their work by using metrics other than expected utility to evaluate risks,^55^ or by including extreme-event scenarios in their analyses.^59^ For example, Baik et al.^56^ and Sepulveda et at.^57^ both highlight how low probability, high cost reliability failures in the electric grid could have substantial effects on the choice of an optimal electricity system.

## Future directions

The critiques of MES research discussed in this paper suggest some gaps and weaknesses in the current strategies employed by the community. In this section, we identify ways in which the MES modeling community can continue to inform decarbonization, environmental health policy, and energy equity policy while addressing these critiques. The priorities explored in this section range from research best practices to methodological improvements.

### Community infrastructure for model sharing and collaboration

Increasingly, the MES community is adopting model sharing best practices, such as code and data sharing, model documentation, and tutorials for researchers and stakeholders.^44^^,^^45^^,^^46^ Community support for model-sharing efforts like the openmod initiative,^48^ TIMES,^73^ and GenX^23^ creates well established and maintained models with steadily improving feature sets, avoiding replicated effort to rebuild the same model. The point is these efforts go beyond sharing code and into establishing communities that build, share, teach, and keep up-to-date common modeling infrastructure. These model-sharing efforts have set the stage for fundamental changes in how researchers present their work, both to the rest of the research community and to stakeholders. More modelers are making their code and data publicly available on platforms like GitHub, improving model transparency and ease of model extension. Although model sharing typically helps speed innovation, it can also lock-in model assumptions, structures, and features. Therefore, as the MES community transitions to publicly available models, they should strike a balance between building on existing work and exploring fundamentally different models. Documentation has also proved an important component of the model and data sharing initiative in the MES community. Documentation allows modelers to discuss their key assumptions, improving clarity for other researchers looking to adopt and extend their models. The complexity and diversity of tools employed by the MES community make live tutorials a valuable extension of written documentation, as the barrier to entry of MES tools can be considerable. Lessons from COVID-era workshops show promising strides toward improved accessibility as the push toward virtual tutorials improves access to all. Finally, this paper itself and the workshop series it stems from^4^ represent ongoing efforts to improve the community infrastructure and collaboration.

### Increased stakeholder involvement

Stakeholders such as policy-makers, utilities, private energy entities, and individuals affected by energy policy play a key role in the commission and consumption of MES research. Many of the critiques raised in this paper and from the broader MES ecosystem stem from insufficient stakeholder involvement in the MES research process. The policy realism critique reflects a disconnect between policies considered in reality and MES research. The model transparency critique can be addressed through resources making MES research more accessible to those outside the research community. Strategies to address the model complexity critique involve a collaboration with stakeholders to include only relevant details. Methodologically, one way of involving stakeholders in the modeling process is through MGA.^69^ MGA can address the complexity, large decision spaces, and competing objectives facing stakeholders. However, work remains to develop methods that present the many potential portfolios produced by MGA in intuitive ways. Outside methodological changes, MES researchers can facilitate stakeholder involvement by expanding the communication channels between researchers and stakeholders. In this way, stakeholders can contribute throughout the research process, including the inception, data acquisition, modeling decisions, interpretation of results, and dissemination of the conclusions.

### Expansion of model boundaries and capabilities

Rapid changes in the energy system landscape are requiring MES researchers to adjust their modeling frameworks. These adaptations fall into four categories: equity, policy realism, treatment of uncertainty, and bridging micro to macro interactions.

#### Consideration of distributional effects

Modelers are increasingly improving model representation of distributional considerations to support decision making beyond a financial efficiency lens.^39^^,^^43^^,^^70^ The models do not exclude cost parameters; they provide the basis for policy alternatives that keep costs at the same or similar level, whereas also prioritizing other important non-cost considerations such as lower emissions and positive impacts on employment. As a result, policymakers are shown the trade-offs between financial efficiency and equity.^69^^,^^70^ One simplified or brute force approach to this type of modeling is one in which the objective is defined as the social, political or equity related objective of concern, whereas the cost is added as a constraint. This produces results that show the maximum benefit possible under a cost constraint. A core challenge with this approach is that quantification can actually be quite limiting and subjective, as discussed in the section titled “Subjective parameters”. In this, the MES modeling community can also learn from practitioners in adjacent MES areas such as the social cost of carbon (SCC), where distributional effects have a more established presence in the literature.

#### Enhancing policy realism

Beyond distributional considerations, MES modelers have also begun to incorporate multiple dimensions of policy realism in their analysis.^35^^,^^67^ Here, there are several possible research areas that may be fruitful. First, MES modelers could borrow tools from political science to systematically evaluate the political feasibility of proposed instruments for decarbonization. Second, MES modelers could draw on insights from psychology and other social sciences to understand and incorporate possible effectiveness of different instruments from a behavioral lens. Lastly, MES modelers could use more granular models with detailed representation of proposed policies to form model recommendations appropriate for their specific decision making context.

#### More thorough treatment of uncertainty

The methodological and computational challenges associated with modeling uncertainty are at the center of this active and growing area of research in the MES community. To address these challenges, modelers can use uncertainty analysis and quantification to assess the range over which their conclusions hold true.^55^ First, some uncertainty in MES models stems from exogenous inputs to the models, such as parameter distributions, costs, and climate forecasts.^31^ In these cases, MES researchers may need to parse out the uncertainty from upstream models before assessing their impact on their own work. Second, perhaps the largest source of uncertainty, the stochastic natural hazards resulting from climate change, can be modeled either by using the outcome of random events, or incorporated structurally in the model as scenarios. These two approaches may result in fundamentally different results and are therefore an important consideration for researchers building MES models. Third, parametric uncertainty can be addressed through sensitivity analysis, revealing the range of inputs over which conclusions remain valid. In addition to sensitivity and scenario analysis, more work could investigate optimal short-term decision making to hedge against future uncertainties and allow for effective recourse decisions down the road (e.g., through stochastic optimization). Finally, improved transparency about model assumptions and limitations helps address concerns about both parametric and structural uncertainty.

#### Bridging micro to macro interactions

Recent changes in energy systems require integrated modeling frameworks to study the interactions between different sectors, particularly end-use sectors, and their effects throughout the rest of the economy. Traditionally, MES researchers have either built bottom-up modeling tools that represent the dynamics within a sector in detail, or used large-scale top-down Computable General Equilibrium (CGE) models or Integrated Assessment Models (IAM) to explore economy-wide impacts of future changes. However, the former approaches may fail to endogenously account for all the interactions between sectors, whereas the latter approaches generally have coarse geospatial, temporal, and technology representations incapable of capturing interactions in the detail at which they occur. For example, as electric vehicles (EVs) diffuse throughout the vehicle fleet, modeling of transportation-energy sector interactions may need to consider the additional electricity demand EV charging will require. However, most IAMs and other macro-scale tools lack the temporal resolution to model the dynamics of EV charging. Similarly, modeling the energy system’s interaction with the human sector requires a level of detail that is typically idealized and abstracted away in large-scale top-down models. Overall, the balance between representing micro-scale inter-sector interactions in macro-scale tools while retaining tractability remains an open question in MES.

## Conclusions

In this section, we first clarify some limitations of this perspective piece then provide a summary of our findings and conclusions.

### Limitations of the study

The conclusions and recommendations provided in this perspective should be interpreted with four limitations in mind. First, some of the critiques referenced in this paper have been applied to research areas that existed before they were unified under the umbrella of MES. These areas, such as energy system and integrated assessment modeling, developed some of their own strategies to address these critiques. Although they began before the term “macro-energy systems” was introduced, we cite some of these efforts as responses to the critiques of MES modeling research in Table 1. Second, although this perspective emphasizes the important contributions that other types of MES researchers can make to efforts to respond to the identified critiques, the critiques themselves are tailored to quantitative modelers in MES. One of the benefits of unifying research under the broad heading of MES is that often a weakness in one area can be addressed with input from researchers who approach MES with a different set of disciplinary tools and ideas. Third, in Table 1 we highlight examples of papers that either raise the specific critique or represent recent or ongoing work to address it. However, the MES literature is vast, so the examples we highlight are not exhaustive; because of space limitations, we omit many excellent studies that are addressing the shortcomings of MES modeling tools. Finally, this paper builds on the collective views of researchers expressed at two recent MES workshop events.^4^ There are surely other important perspectives that are not represented by this subset of the MES community. Our goal is not to exclude any perspectives beyond those reflected in this paper, but rather to invite researchers to join the conversation.

### Summary

The emergence of MES as a unified field represents the merging of previously siloed researched areas all aimed at addressing the same overarching research question: how to equitably and economically satisfy the world’s energy needs while combating climate change and other environmental threats.^2^ This paper aims to advance the growth of the field by delineating common critiques, summarized in Table 1, that have the potential to slow progress. This paper evaluates the validity of these critiques, discusses ongoing research that addresses them, and identifies areas that represent the frontiers of MES research. The interdisciplinary nature of MES makes explicit enumeration and discussion of critiques and research priorities critical for effective communication within the MES research community. By identifying these critiques and discussing current and future efforts to address them, this paper aims to establish a clear path forward for the MES community.

Critiques of MES research fall into three main categories. First, there are critiques of the data collection and model validation processes. These critiques capture challenges that are inherent to MES research, stemming from fundamental limitations because of the forward-looking nature of MES models, or else rapid change of parameters exogenous to MES models. Methods for addressing these critiques focus on model evaluation, such as sensitivity analysis, as well as model transparency. Second, some critiques target MES model structure and assumptions such as modeling of tail risks and the systemic focus on parametric rather than structural uncertainty. MES researchers are addressing this class of critiques through increased model transparency, particularly by involving stakeholders and researchers in adjacent fields more deeply in the modeling process, and by broadening the range of scenarios included in their work. Finally, the third class of critiques of the MES field challenge the focus and boundaries of MES models. These critiques question the realism, level of complexity, and equity of MES models. Again, better communication between MES researchers and stakeholders helps improve model realism and highlight the potential limitations of the work to those interpreting results for guidance in policy decisions, clearly establishing a boundary between MES research and policy recommendations. Expansion of MES models to include more diverse modeling mechanisms and objective functions also helps address these critiques.

The frontiers of MES research follow from the ongoing efforts to address critiques of the field. First, developing better community infrastructure for sharing models is pivotal to the progress of the MES field, through data and code sharing and through improved documentation and tutorials to those within and outside the MES community. Second, better communication with stakeholders in MES research will help MES research be more relevant, accurate, and utilized. Finally, MES research will also explore extending their models to include a greater diversity of objectives such as equity, environmental impacts, and political expediency. In addition, more thorough treatment of uncertainty and improved transparency about assumptions in the modeling of uncertainty, in tandem, are increasingly significant model features. In these expanded models, MES will also leverage its diversity as a field, incorporating a wide variety of sectoral considerations, within and beyond those of the energy sector. Overall, the MES research community is making significant progress in addressing existing critiques, and will continue to improve its capabilities by drawing on a growing body of MES experts from diverse disciplinary backgrounds.
